# SMAD4 regulates the progression of cholangiocarcinoma by modulating the expression of STING1

**DOI:** 10.1111/jcmm.17857

**Published:** 2023-07-24

**Authors:** An‐da Shi, Li‐ming Zhao, Guo‐li Sheng, Ge‐ning Zhang, Yong‐chang Tang, Kang‐shuai Li, Zong‐li Zhang

**Affiliations:** ^1^ Department of General Surgery, Qilu Hospital, Cheeloo College of Medicine Shandong University Jinan China; ^2^ Master of Public Health The University of Queensland Brisbane Queensland Australia

**Keywords:** cholangiocarcinoma, immunogenicity, immunotherapy, SMAD4, STING1

## Abstract

SMAD4 is a tumour suppressor and an important regulator of tumour immune scape which is downregulated in cholangiocarcinoma (CCA). STING1 is a vital sensing factor of abnormal DNA; however, the correlation between SMAD4 and STING1 and the role of the SMAD4‐STING1 interaction in the progression of CCA have not yet been evaluated. Public database was analysed to reveal the expression of SMAD4 and STING1. A cohort comprising 50 iCCA, 113 pCCA and 119 dCCA patients was assembled for the study. Immunohistochemistry was employed to evaluate the expression levels of STING1 and SMAD4. In vitro transwell and CCK8 assays, along with luciferase reporter assay, were conducted to analyse the potential regulatory mechanisms of SMAD4 on the expression of STING1. Expression of SMAD4 and STING1 were downregulated in CCA tumours and STING1 expression correlated with SMAD4 expression. The overexpression of SMAD4 was found to suppress the migration, invasion and proliferation capabilities of CCA cells; whereas, the knockdown of SMAD4 enhanced these abilities. Furthermore, it was observed that SMAD4 translocated into the nucleus following TGF‐β1 stimulation. Knockdown of SMAD4 resulted in the inhibition of STING1 transcriptional activity, whereas the overexpression of SMAD4 promoted the transcriptional activity of STING1. Clinically, low STING1 and SMAD4 expression indicated poor prognosis in CCA, and simultaneously low expression of STING1 and SMAD4 predicts poorer patient survival. SMAD4 regulates the expression of STING1 through its transcription regulating function. Dual low expression of STING1 and SMAD4 had more power in predicting patient survival. These results indicate that SMAD4‐silenced CCA may downregulate its STING1 expression to adapt to the immune system.

## 1 INTRODUCTION

Cholangiocarcinoma (CCA) consists a group of malignant tumours originating from the bile duct epithelium.^1^, ^2^, ^3^ CCA, a type of bile duct cancer, is classified into two main subtypes based on its anatomical location: intrahepatic CCA (iCCA) and extrahepatic CCA (eCCA). The eCCA subtype can be further divided into perihilar CCA (pCCA) and distal CCA (dCCA) based on its specific location within the bile ducts.^4^, ^5^, ^6^ CCA differs from other malignant hepatobiliary tumours as CCA contains more genomics variations.^7^ KRAS, TP53, IDH1/2 and ARID1A are the most common mutated genes in iCCA^8^ while KRAS, TP53, ARID1A and SMAD4 are the most common mutated genes in eCCA.^9^ despite the presence of numerous actionable genomic alterations in CCA, only a limited number of established treatment regimens are available for this disease.^5^, ^7^ For advanced CCA, the first‐line treatment is gemcitabine‐based chemotherapy, while for patients with FGFR fusion mutation, the optional treatment scheme includes pemigatinib and infigratinib.^10^, ^11^, ^12^ A significant progress has been achieved in the treatment of advanced stage CCA patients through the utilization of immunotherapy. Despite the high immunogenicity of CCA, the effectiveness of immunotherapy in treating this disease has been limited. Therefore, it is essential to delve deeper into the underlying reasons behind the suboptimal therapeutic outcomes and explore potential strategies for improving immunotherapy in CCA.^13^, ^14^

In our previous study, we found that the expression of SMAD4 was suppressed in iCCA and pCCA, and the expression of SMAD4 was positively correlated to the prognosis of CCA.^15^ SMAD4 is a tumour suppressor and participates in the regulation of the transforming growth factor β (TGF‐β) signalling pathway.^16^, ^17^, ^18^ Its absence can lead to the occurrence of gastrointestinal tumours. The loss of SMAD4 has been correlated with a poorer prognosis and an increased likelihood of chemotherapy resistance.^19^ The deletion of SMAD4, often observed in advanced tumour stages and lymph node involvement, is typically accompanied by decreased peritumoral lymphocyte aggregation and a lower presence of tumor‐infiltrating lymphocytes (TILs). Additionally, the loss of SMAD4 has been associated with chromatin instability, further emphasizing its potential role in CCA progression.^18^ However, low expression of SMAD4 is often associated with poor response to immunotherapy, and its mechanism is worthy of further exploration.^17^, ^20^, ^21^, ^22^

cGAS‐STING1 is a vital sensing factor of abnormal DNA in cells.^23^, ^24^ The inherent DNA instability in tumour cells can stimulate their intrinsic cGAS (cyclic GMP‐AMP synthase) to generate cGAMP, thereby activating the immune response and ultimately resulting in the elimination or clearance of the tumour cells.^25^, ^26^, ^27^, ^28^ However, the promoter regions of cGAS and STING1 in tumour cells are usually highly methylated and silenced or have a loss of function mutation. The expression and potential role of STING1 in the progression of CCA have not been elucidated.

In this study, we conducted an analysis of STING1 and SMAD4 expression in both CCA tumour tissues and adjacent normal tissues. Furthermore, we investigated the potential correlation between the expressions of STING1 and SMAD4. Furthermore, we examined the prognostic implications of STING1 expression and the co‐expression of STING1 and SMAD4.

## 2 MATERIALS AND METHODS

### 2.1 CCA cohort

The cohort containing 50 iCCA patients, 113 pCCA patients, and 119 dCCA patients (Table S1–S3) was established as previously described. The tumours were classified and staged in accordance with the eighth edition of the AJCC/UICC TNM classification system. The Ethics Committee of Qilu Hospital, Shandong University has approved this study and all patients involved in this study provided informed consent.

### 2.2 Tissue microarrays and IHC

The tissue microarray (TMA) incorporated tissue sections that were preserved through formalin fixation and paraffin embedding. Haematoxylin and eosin staining was then performed to analyse the histological attributes of the samples. Each sample, measuring 1.5 mm in diameter, was orderly arranged onto slides.

For immunohistochemistry (IHC), slides were deparaffinized and pre‐treated in EDTA buffer (pH 9.0). Primary anti‐STING1 antibody (Abcam, ab239074, 1:1000) or anti‐SMAD4 antibody (Abcam, ab40759, 1:100) was applied and incubated at 4°C overnight. Goat anti‐rabbit antibody (Zsbio) was applied at room temperature for 30 min. Then slides were incubated with 3,3‐diaminobenzidine (DAB) solution (Zsbio).

The IHC score of tumour areas was quantified by QuantCenter software.^29^ H score method was performed to analyse the expression of STING1 and SMAD4. The cut‐off point was determined by the receiver operating characteristic (ROC) curve according to a previous study.^30^

### 2.3 Cells and reagents

The human CCA cell lines, QBC‐939 and RBE, were acquired from the Cell Bank of the Chinese Academy of Sciences, with their authenticity confirmed through STR analysis. RBE cells were cultured in RPMI‐1640 medium (Gibco), while QBC‐939 cells were cultured in DMEM (Gibco) supplemented with 10% fetal bovine serum (FBS, Thermo Fisher) and 1% penicillin/streptomycin. All cells were cultured in a thermostatic cell incubator with a 5% CO_2_ atmosphere.

Main reagents and antibodies applied are listed below:

**Table:** 

Antibodies	Source	Identifier
STING1	Abcam	Cat. No. ab239074
SMAD4	Abcam	Cat. No. ab40759
TGF‐βR2	Abcam	Cat. No. ab270440
Histone H3	Immunoway	Cat. No. YM3038
GAPDH	Santa Cruz	Cat. No. sc‐47724

Recombinant proteins		
TGF‐β1	Abcam	Cat.No. ab50036

### 2.4 RNA extraction and quantitative real‐time PCR

RNAs were extracted from the CCA cell lines by TRIzol (Thermo Fisher) according to the manufacturer's manual. The reverse transcriptase kit (Roche), SYBR Green Master Mix (Yeasen), and a Light Cycler Roche 480 PCR instrument was applied to synthesize cDNA and perform the quantitative real‐time PCR (qPCR). Relative expression of SMAD4 was calculated using the 2^−ΔΔCt^ method, and β‐catenin was adopted as an internal control. The sequence of primers used are as follows:

**Table:** 

Name	Primer sequence (5′–3′)
SMAD4	F: CCAATCATCCTGCTCCTGAGT R:CCAGAAGGGTCCACGTATCC
β‐catenin	F: GCTGCAACTAAACAGGAAGGG R: CCCACTTGGCAGACCATCAT

### 2.5 Western blotting

Cells were lysised in RIPA lysis buffer (Solarbio) supplemented with 1% PMSF and 1% phosphatase inhibitor on ice for 30 min. Cell membrane proteins and plasma proteins were extracted by cell membrane protein extraction kit (Sigma‐Aldrich). After WB electrophoresis and transmembrane, the PVDF membranes were then incubated with the primary antibodies at certain dilution (1:1000 for SMAD4, 1:1000 for Histone H3, or 1:1000) for GAPDH overnight at 4°C, and then incubated with the secondary antibodies (1:5000) for 1 h. The protein bands are visualized by enhanced chemiluminescence.

### 2.6 Transfection of cells

siRNAs or scramble oligos (GenePharma) were transfected with RNAiMAX for transient knockdown of SMAD4. The target sequences of siRNAs are listed in the following table:

**Table:** 

Name	The target sequence of siRNA (5′–3′)
siSMAD4	AAGGTGGAGAGAGTGAAACAT
scramble‐siSMAD4	TTCTCCGAACGTGTCACGT

### 2.7 CCK8 assay

CCA cells transfected with siRNA/overexpression sequence were plated into 96‐well plates (2 × 10^3^ cells/well) and incubated for 1–5 days. During 24‐h‐interval, 10 μL of CCK‐8 reagent (Yeasen) was added into each well and incubated with the cells at 37°C for 30 min. Then the cell number was measured through absorbance value at 450 nm which was detected using a spectrophotometer.

### 2.8 Transwell assay

The upper chamber (8.0 μm in pore size; Corning) was loaded with Matrigel (BD Biosciences) before plating cells for the invasion experiments. 2–5 × 10^5^ CCA cells were plated into each upper chamber either with or without matrigel. Culturing medium containing 20% FBS was added to the lower chambers to induce the migration or invasion of CCA cells. After incubation for 24 h, the cells stayed on the upper surface were wiped off and the cells migrated to the lower surface were stained with 0.5% crystal violet (Beyotime) for 30 min. Cell number was counted from five randomly selected visual felds with a microscope.

### 2.9 mRNA‐seq

After transfecting SMAD4 knockdown or scramble plasmid in CCA cell line QBC‐939, total RNA was extracted using Trizol reagent (Invitrogen) following the manufacturer's procedure. The total RNA quantity and purity were analysis of Bioanalyzer 2100 and RNA 6000 Nano LabChip Kit (Agilent) with RIN number >7.0. Approximately 10 μg of total RNA representing a specific adipose type was subjected to isolate poly(A) mRNA with poly‐T oligoattached magnetic beads (Invitrogen). Following purification, the poly(A)− or poly(A)+ RNA fractions are fragmented into small pieces using divalent cations under elevated temperature. Then the cleaved RNA fragments were reverse transcribed to create the final cDNA library in accordance with the protocol for the mRNA‐Seq sample preparation kit (Illumina), the average insert size for the paired‐end libraries was 300 bp (±50 bp). And then we performed the paired‐end sequencing on an Illumina Novaseq™ 6000 at the (lc‐bio) following the vendor's recommended protocol.

### 2.10 Immunofluorescence assay

Cells were initially seeded on chamber slides, which were then placed in 24‐well plates and allowed to incubate overnight. Following incubation, the cells were carefully washed with PBS and subsequently fixed with 4% paraformaldehyde at room temperature for a duration of 20 min. After an additional PBS wash, the cells were subjected to a blocking/permeabilization buffer (composed of 10% normal goat serum and 0.2% saponin in PBS) at room temperature for 30 min. Subsequently, the cells were incubated in an antibody dilution buffer (consisting of 10% normal goat serum and 0.05% saponin in PBS) at room temperature for 60 min, followed by an overnight incubation with primary antibodies targeting TGF‐βR2 (1:200) and SMAD4 (1:200). Finally, the cells were exposed to Alexafluor‐conjugated secondary antibodies (1:50) at room temperature for 60 min.

### 2.11 Luciferase reporter assay

The promoter region sequence of STING1 was predicted by transcription‐factor‐predicting software (Jaspar). Then the promoter regions were amplified by PCR and then cloned into the PGL3‐basic dual luciferase reporter plasmid through the KpnI/HindIII sites to generate STING1 luciferase reporters. The QBC‐939 cells with stable transfected siSMAD4 or overexpression plasmid were seeded with suitable concentration in 24‐well plates in triplicate. Then the preconditioning cells were transfected with the indicated plasmids and pRL‐TK Renilla plasmid using Lipofectamine 3000 (Thermo Fisher Scientific). After 48 h, luciferase activity and Renilla signals were detected using a DLR Assay Kit (Promega, Madison) according to the instructions. The activity of the reporter gene was assessed by normalizing the firefly luciferase activity against that of the Renilla luciferase. The promoter region sequences (5′‐3′) of STING1 was listed as following: STING1 promoter region spanning nucleotides −849 to −859 TGATTTGATGC.

### 2.12 Statistical analysis

Statistical evaluations were conducted using SPSS 26.0 and GraphPad Prism 8.1 software. The correlation between STING1 or SMAD4 expression and clinicopathological characteristics was analysed with Chi‐squared test. The survival curves were drawn with Kaplan–Meier method and the difference between the two groups was compared with the logrank test. Multivariate analysis was performed with the Cox proportional hazards regression model to determine the independent prognostic significance of clinicopathological characteristics. The statistical differences between groups were calculated using one‐way anova or *t*‐test. *p* < 0.05 was considered significant.

## 3 RESULTS

### 3.1 Expression of STING1 and SMAD4 was downregulated in CCA

**FIGURE 1:**
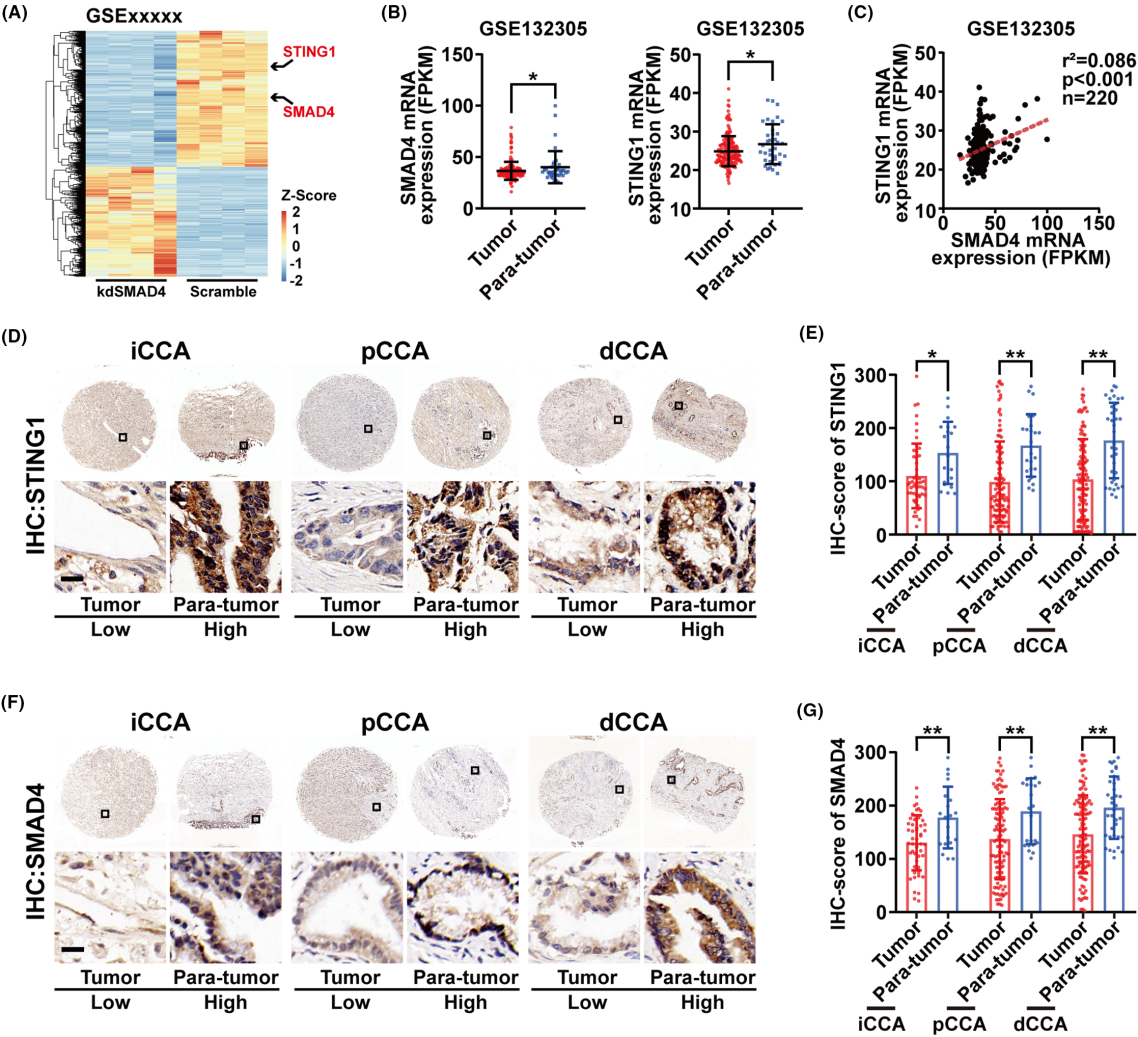
The expression of STING1 and SMAD4 was downregulated in cholangiocarcinoma (CCA). (A) The results of mRNA‐seq in QBC‐939 cell line showed that the mRNA expression of STING1 decreased with SMAD4 knockdown (GSE236894). (B) The analysis of mRNA expression for STING1 (right) and SMAD4 (left) in the GSE132305 database is displayed. *p < 0.05 (C) A correlation analysis between the mRNA expression of SMAD4 and STING1 was conducted. (D) Provided are representative images of immunohistochemical staining for STING1 in iCCA (left), pCCA (middle), and dCCA (right) using the tissue microarray. (E) The expression of STING1, as evaluated by the IHC score, in paired tumour and adjacent normal tissues is examined. (F) Representative images of immunohistochemical staining for SMAD4 in iCCA (left), pCCA (middle), and dCCA (right) from the tissue microarray are displayed. (G) The expression of SMAD4, evaluated based on the IHC score, in paired tumour and adjacent normal tissues is analysed. *p < 0.05; **p < 0.01.

To analyse the expression level of STING1 and SMAD4 and their correlation, we performed SMAD4 knockdown on CCA cell line QBC‐939, followed by transcriptome high‐throughput sequencing (GSE236894). And the results showed that the expression of STING1 decreased with SMAD4 knockdown (Figure 1A). Then we analysed the mRNA expression of STING1 and SMAD4 in the public database. We explored a database containing genome‐wide expression data of 182 eCCA and 38 non‐tumoral bile duct samples. The result revealed that the mRNA expression level of SMAD4 and STING1 was significantly downregulated in eCCA tumour tissues compared to the para‐tumour tissue. Meanwhile, the expression of STING1 was positively correlated with the expression of SMAD4 (*p* < 0.001) (Figure 1B,C). To further analyse the expression of STING1 and SMAD4 on the protein level, we constructed a tissue microarray (TMA) containing 50 paired tumour and para‐tumour tissues of iCCA, a tissue microarray containing 113 paired tumour and para‐tumour tissues of pCCA and a tissue microarray containing 119 paired tumour and para‐tumour tissues of dCCA. Immunohistochemistry (IHC) was then performed to analyse the protein expression of STING1 and SMAD4. As shown in Figure 1D–G, STING1 and SMAD4 were expressed mainly in intracellular space. Expression of STING1 and SMAD4 were downregulated in tumour tissues of iCCA, pCCA and dCCA compared to the para‐tumour tissue.

### 3.2 STING1 expression positively correlated with SMAD4 expression in CCA

**FIGURE 2:**
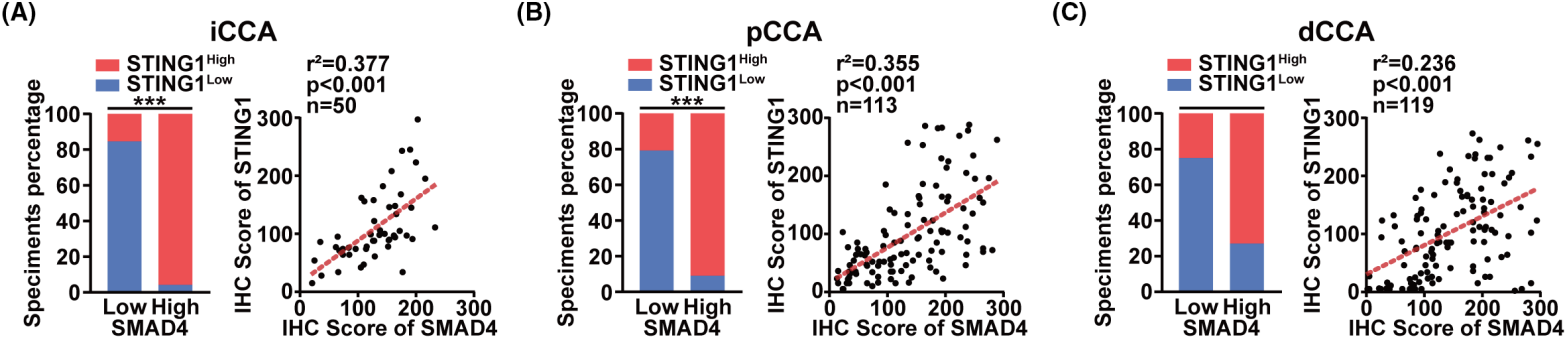
STING1 expression correlated with SMAD4 expression. (A) An analysis was conducted to assess the correlation between SMAD4 and STING1 protein expression in intrahepatic cholangiocarcinoma (iCCA). (B) The correlation between SMAD4 and STING1 protein expression in perihilar CCA (pCCA) was examined. (C) The study also evaluated the correlation between SMAD4 and STING1 protein expression in distal CCA (dCCA).

Correlation analysis at the mRNA level revealed a positive correlation between the expression of STING1 and SMAD4. We further evaluated this relationship at the protein level using our tissue microarray (TMA). As depicted in Figure 2, STING1 expression positively correlated with SMAD4 expression in intrahepatic CCA (iCCA; *r* ^2^ = 0.377, *p* < 0.001), perihilar CCA (pCCA; *r* ^2^ = 0.355, *p* < 0.001) and distal CCA (dCCA; *r* ^2^ = 0.236, *p* < 0.001).

### 3.3 SMAD4 overexpression inhibited CCA cell migration, invasion and proliferation while knockdown of SMAD4 promoted the migration, invasion and proliferation ability of CCA cells

**FIGURE 3:**
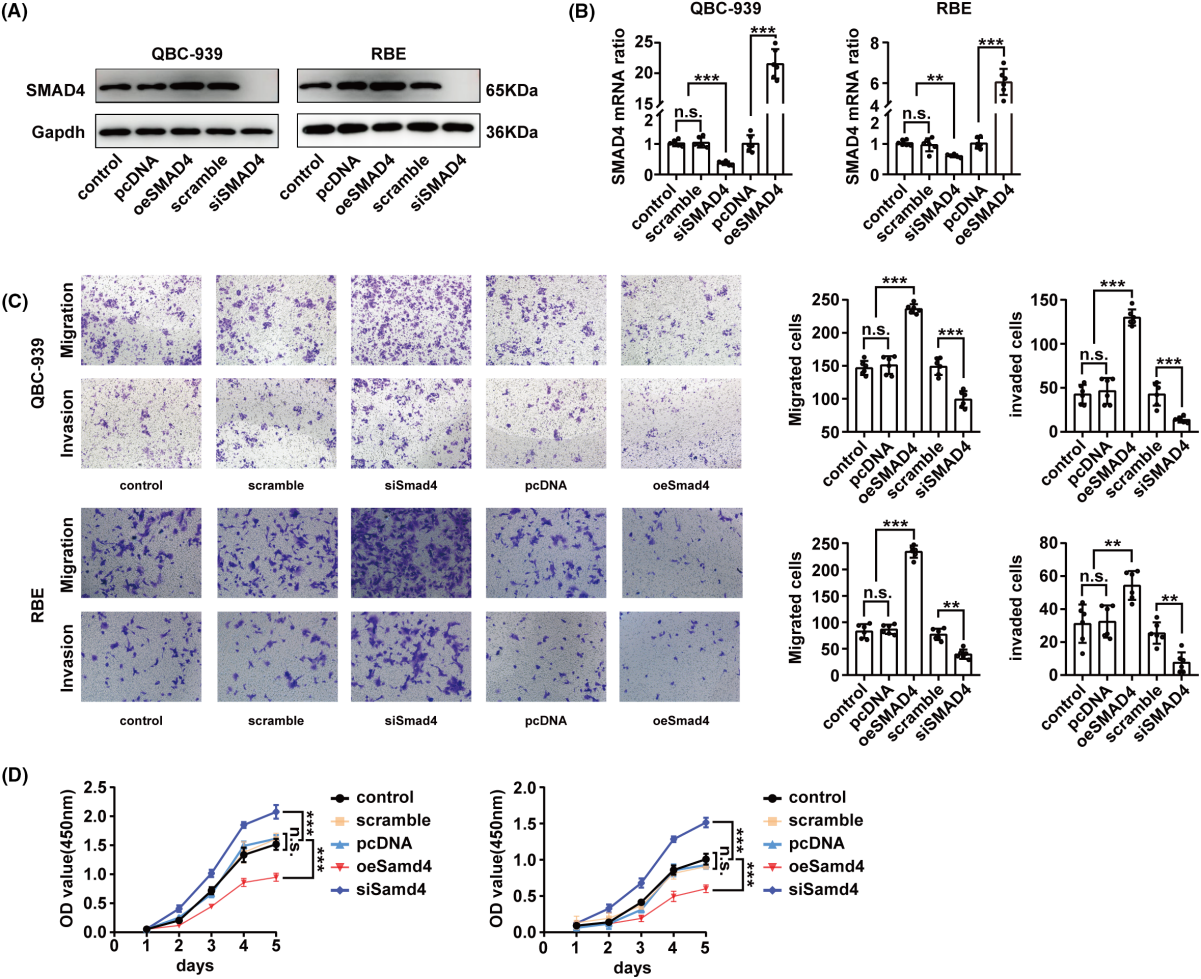
Effect of SMAD4 knockdown and overexpression on the migration, invasion, and proliferation ability of cholangiocarcinoma (CCA) cells. (A,B) Successful knockdown and overexpression of SMAD4 in QBC‐939 and RBE cells were confirmed by western blot (A) and qPCR (B). (C) The migration and invasion of SMAD4‐overexpressed or ‐silenced CCA cells were examined using transwell assays (right panel). Representative images of the transwell assays are shown in the left panel. Scale bar: 100 μm. (D) The proliferation of SMAD4‐overexpressed or ‐silenced CCA cells was assessed with the CCK8 assay. In figures B–D, ‘n.s.’ denotes not significant. *, **, and *** represents *p*‐values <0.05, <0.01, and <0.001, respectively. Statistical significance was analysed using one‐way anova in panels B and C (right) or two‐way anova in panel D. Data are derived from at least three independent experiments and are represented as mean ± SEM.

Given the observed downregulation of SMAD4 expression in CCA, we further explored the mechanism by which SMAD4 impacts the progression of CCA. We established SMAD4‐overexpression and SMAD4‐knockdown cell lines in the QBC‐939 and RBE cell lines. The efficiencies of overexpression and knockdown were confirmed using western blot and qPCR (Figure 3A,B). Transwell migration and invasion assays demonstrated that the migration and invasion capabilities were enhanced in SMAD4‐knockdown QBC‐939 and RBE cells compared to control cells. In contrast, these capabilities were significantly reduced in SMAD4‐overexpressing cells when compared to the scramble cells (Figure 3C). A CCK8 assay was conducted to evaluate the impact of SMAD4 overexpression on CCA cell proliferation (Figure 3D). It was found that SMAD4 upregulation inhibited the proliferation of both QBC939 and RBE cell lines in vitro, whereas SMAD4 downregulation promoted cell proliferation in both cell lines.

### 3.4 TGF‐β1 stimulation promoted the nucleus translocation of SMAD4 and SMAD4 regulated the transcription activity of STING1

**FIGURE 4:**
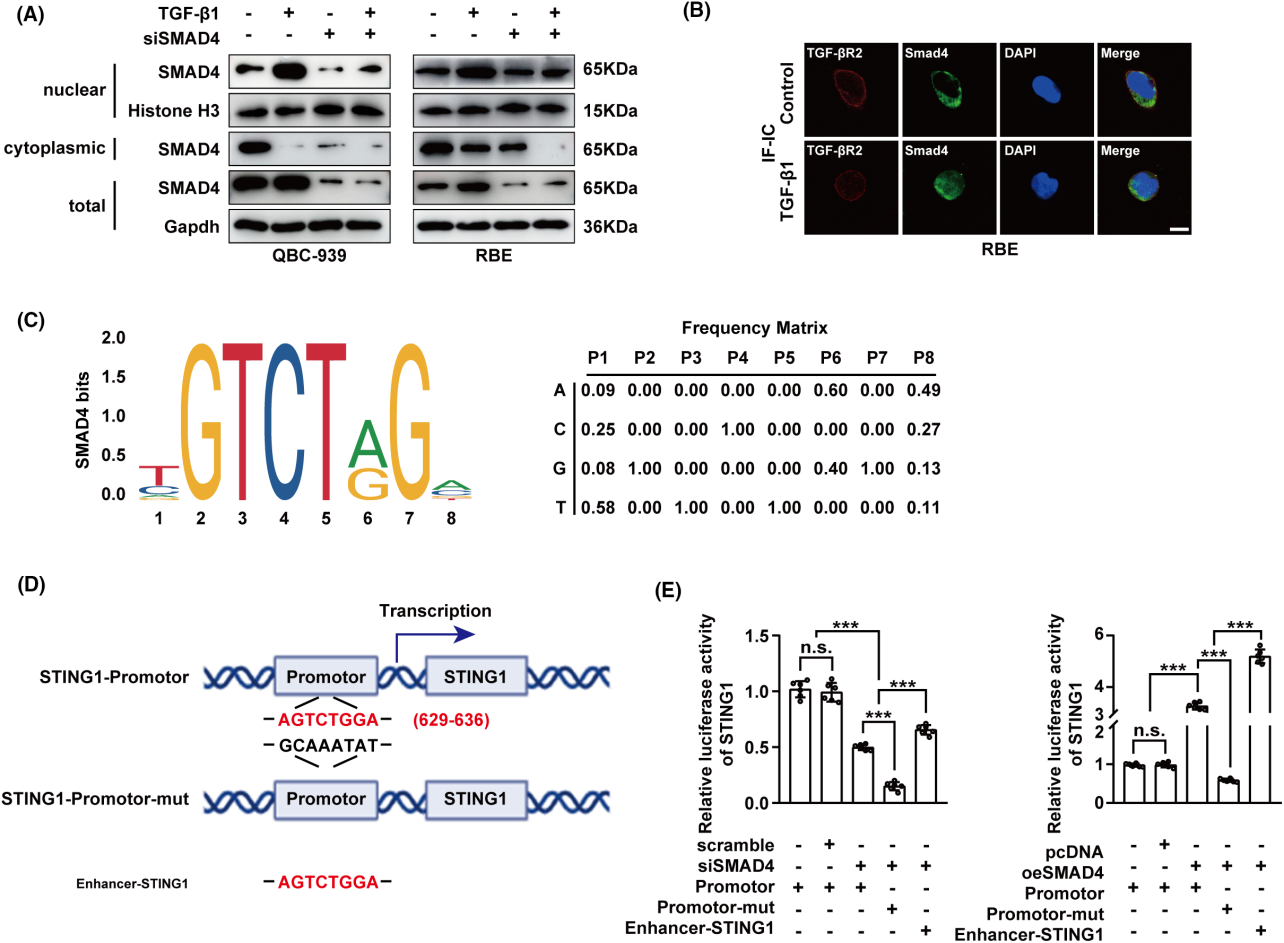
SMAD4 enters nucleus after TGF‐β1 stimulation and regulates STING1 transcription activity. (A,B) Detected with WB (A) and IF (B) of CCA cells in the presence/absence of TGF‐β1 or siRNAs of SMAD4. SMAD4 could transport into the nucleus by TGF‐β1 stimulation. Scale bar: 20 μm. (C) By Jaspar, the promoter region sequence of STING1 was predicted (left panal). The nucleobase frequency matrix was listed as right panal. (D) STING1 promoter region spanning nucleotides was −849 to −859 TGATTTGATGC. (E) By detected with diluciferase assay, SMAD4 regulates transcription of STING1 by transporting into the nucleus. In (E), ‘n.s.’ denotes not significant, while *, **, and *** represents *p*‐values <0.05, <0.01, and <0.001, respectively. Statistical significance was evaluated using one‐way anova. The data are derived from at least three independent experiments and are expressed as mean ± SEM.

Given the observed correlation between SMAD4 and STING1 expression in CCA, we further delved into the mechanism by which SMAD4 regulates STING1 expression. Considering SMAD4 is a downstream signalling molecule of TGF‐β1, we initially analysed whether TGF‐β1 stimulation altered the intracellular localization of SMAD4. This analysis was performed using a cell component isolation assay and SMAD4 immunofluorescence. As shown in Figure 4A, TGF‐β1 significantly decresed cytoplasmic SMAD4 and increased nuclear SMAD4. Immunofluorescence also showed nucleus translocation of SAMD4 after TGF‐β1 stimulation. We then analysed whether SMAD4 regulates the expression of STING1 through transcriptional regulation. The promoter region sequence of STING1 was predicted by Jaspar and the nucleobase frequency matrix was listed. (Figure 4C) The STING1 promoter region spanning nucleotides was −849 to −859 TGATTTGATGC. (Figure 4D) As shown in Figure 4E, knockdown of SMAD4 significantly downregulated the reporter activity, STING1 promoter mutant exert even further downregulated the reporter activity while enhancer of STING1 reversed partially of the reporter activity. Overexpression of SMAD4 significantly upregulated the reporter activity, overexpression of SMAD4 synergized with enhancer of STING1 in enhancing the reporter activity.

### 3.5 Low STING1 and SMAD4 indicated poor prognosis in CCA, and simultaneously low expression of STING1 and SMAD4 predicts poorer patient survival

**FIGURE 5:**
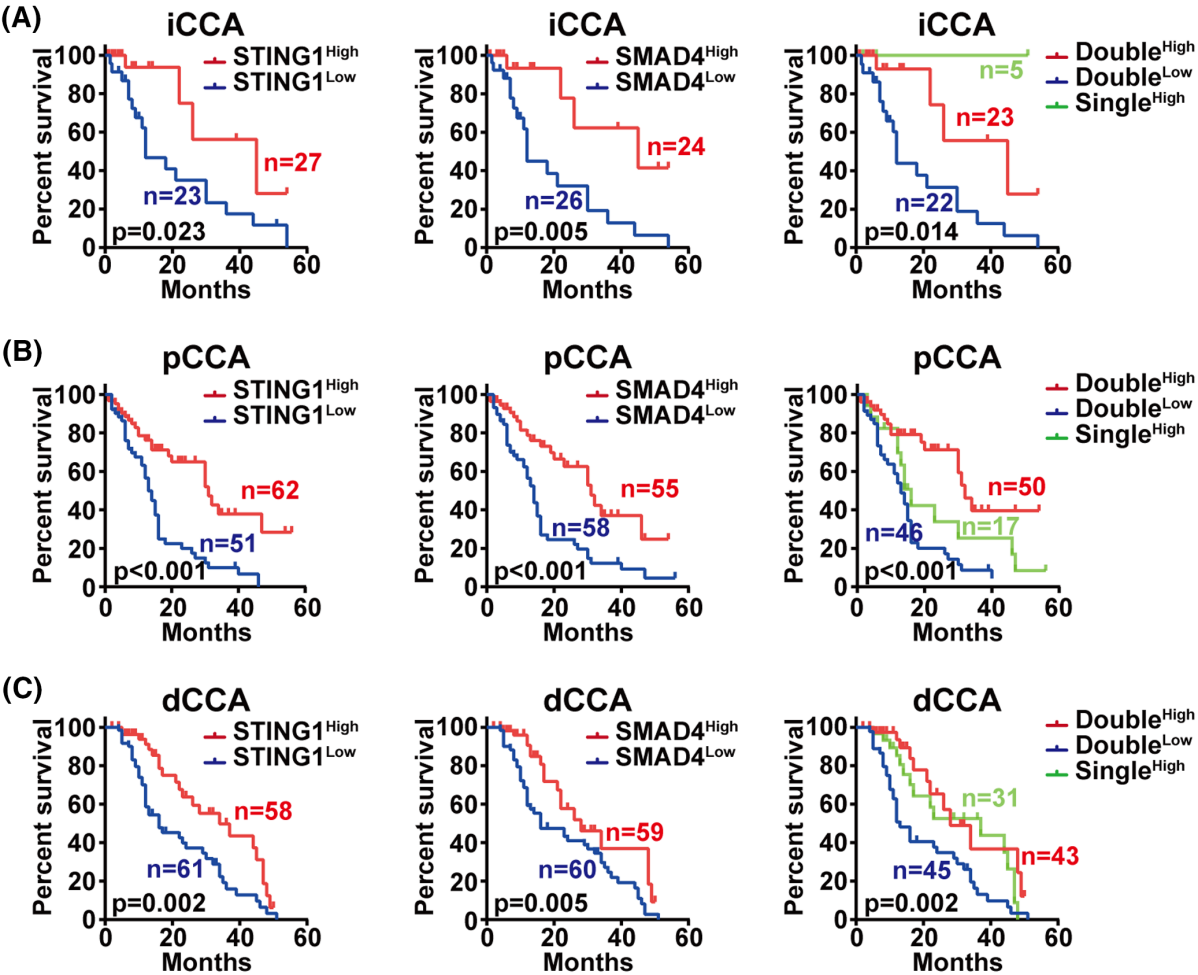
Prognostic role of STING1, SMAD4 and double expression of STING1 and SMAD4 in predicting cholangiocarcinoma (CCA) prognosis. (A) The overall survival curves of patients with intrahepatic CCA (iCCA) are stratified based on STING1, SMAD4 and dual expression of STING1 and SMAD4. Low expression of STING1 and SMAD4 suggests poor overall patient survival. A combination of low expression for both STING1 and SMAD4 is even more predictive of prognosis in iCCA. (B) The overall survival curves of patients with perihilar CCA (pCCA) are stratified by STING1, SMAD4, and the joint expression of STING1 and SMAD4. Low expression of STING1 and SMAD4 indicates poor overall patient survival. Dual low expression of STING1 and SMAD4 proves more effective in predicting patient prognosis in pCCA. (C) The survival curves of patients with distal CCA (dCCA) are stratified by STING1, SMAD4, and the combined expression of STING1 and SMAD4. Low expression of STING1 and SMAD4 signifies poor overall patient survival. Dual low expression of STING1 and SMAD4 is more indicative of patient prognosis in dCCA.

Given the identified correlation between STING1 and SMAD4, we further examined the prognostic role of STING1 and SMAD4 expression in our patient cohort. As depicted in Figure 5A, low STING1 expression correlates with poor prognosis in intrahepatic CCA (iCCA; left, *p* = 0.023), while low SMAD4 expression also correlates with poor patient prognosis (middle, *p* = 0.005). We also assessed the combined effect of STING1 and SMAD4 co‐expression on patient prognosis. As illustrated in Figure 5A, patients with dual‐negative expression of STING1 and SMAD4 exhibited worse prognoses in iCCA. Figure 5B shows a similar pattern in perihilar CCA (pCCA), with low STING1 expression correlating with poor prognosis (left, *p* < 0.001), and low SMAD4 expression also linked to poor patient prognosis (middle, *p* < 0.001). In pCCA, patients with double‐negative expression of STING1 and SMAD4 again showed poorer prognoses. We observed analogous results in distal CCA (dCCA) as low STING1 expression correlated with poor prognosis (left, *p* = 0.002) and low SMAD4 expression also correlated with poor prognosis (middle, *p* = 0.005). Patients with dual‐positive expression of STING1 and SMAD4 exhibited even worse prognosis in dCCA (*p* = 0.002, Figure 5C). These findings suggest that STING1 and SMAD4 work synergistically in the context of CCA.

### 3.6 Expression of STING1 and SMAD4 correlates with none of the known clinicopathological factors

The associations between STING1 expression and the clinicopathological characteristics of CCA were further examined using the Chi‐squared test (Table 1). Expression of STING1 was found to correlate with SMAD4 in intrahepatic (iCCA), perihilar (pCCA) and distal (dCCA) cholangiocarcinomas. However, none of the well‐known risk factors, such as age, gender, tumour size, tumour differentiation, T stage, N stage, M stage, TNM stage, neural invasion and cancer thrombus, were found to correlate with STING1 expression in iCCA and pCCA. Interestingly, high STING1 expression was associated with advanced age in dCCA.Similarly, the associations between SMAD4 expression and the clinicopathological characteristics of CCA were also evaluated (Table 2). None of the established risk factors, including age, gender, tumour size, tumour differentiation, T stage, N stage, M stage, TNM stage, neural invasion and cancer thrombus, were found to correlate with SMAD4 expression in iCCA, pCCA and dCCA.

**TABLE 1:** Correlations between STING1 expression and clinicopathological characteristics in CCA.

Characteristics	Category	iCCA		*p*	pCCA		*p*	dCCA		*p*
		STING1−(*n* = 23)	STING1 + (*n* = 27)		STING1−(*n* = 54)	STING1 + (*n* = 59)		STING1−(*n* = 56)	STING1 + (*n* = 63)	
Age (years)	<60	10	13	0.741	13	22	0.253	25	13	0.030
	≥60	13	14		38	40		36	45	
Gender	Female	6	9	0.577	15	23	0.390	16	20	0.327
	Male	17	18		36	39		45	38	
Tumour size (cm)	<2.5	4	8	0.313	35	49	0.208	39	35	0.686
	≥2.5	19	19		16	13		22	23	
Differentiation	Well/Moderate	15	20	0.496	39	53	0.220	54	52	0.843
	Poor	8	7		12	9		7	6	
T stage	T1/T2	22	25	0.650	28	41	0.223	14	14	0.879
	T3/T4	1	2		23	21		47	44	
N stage	N0	15	23	0.099	33	38	0.708	50	47	0.896
	N1/N2	8	4		18	24		11	11	
M stage	M0	21	24	0.777	49	62	0.116	61	56	0.144
	M1	2	3		2	0		0	2	
TNM stage	I	12	17	0.441	31	36	0.770	51	45	0.406
	II‐IV	11	10		20	26		10	13	
Neural invasion	Negative	16	20	0.723	26	21	0.066	26	19	0.267
	Positive	7	7		25	41		35	39	
Cancer thrombus	Negative	7	7	0.723	15	25	0.227	21	26	0.246
	Positive	16	20		36	37		40	32	
SMAD4	Low	22	4	< 0.001	46	12	< 0.001	45	15	< 0.001
	High	1	23		5	50		16	43	

Abbreviations: CCA, cholangiocarcinoma; dCCA, distal CCA; iCCA, intrahepatic CCA; pCCA, perihilar CCA.

**TABLE 2:** Correlations between SMAD4 expression and clinicopathological characteristics in CCA.

Characteristics	Category	iCCA		*p*	pCCA		*p*	dCCA		*p*
		SMAD4 −(*n* = 27)	SMAD 4 + (*n* = 23)		SMAD 4−(*n* = 55)	SMAD 4 + (*n* = 58)		SMAD 4−(*n* = 51)	SMAD 4 + (*n* = 68)	
Age (years)	<60	13	10	0.555	16	19	0.424	23	15	0.131
	≥60	13	14		42	36		37	44	
Gender	female	7	8	0.621	17	21	0.318	15	21	0.208
	male	19	16		41	34		45	38	
Tumour size (cm)	<2.5	5	7	0.411	44	40	0.703	38	36	0.794
	≥2.5	21	17		14	15		22	23	
Differentiation	Well/Moderate	17	18	0.459	44	48	0.119	52	54	0.396
	Poor	9	6		14	7		8	5	
T stage	T1/T2	25	22	0.504	33	36	0.351	14	14	0.959
	T3/T4	1	2		25	19		46	45	
N stage	N0	17	21	0.067	36	35	0.863	49	48	0.965
	N1/N2	9	3		22	20		11	11	
M stage	M0	23	22	0.706	56	55	0.165	60	57	0.150
	M1	3	2		2	0		0	2	
TNM stage	I	12	17	0.077	34	33	0.881	52	44	0.095
	II‐IV	14	7		24	22		8	15	
Neural invasion	Negative	18	18	0.650	27	20	0.272	26	19	0.211
	Positive	8	6		31	35		34	40	
Cancer thrombus	Negative	8	6	0.650	21	19	0.854	23	24	0.794
	Positive	18	18		37	36		37	35	

Abbreviations: CCA, cholangiocarcinoma; dCCA, distal CCA; iCCA, intrahepatic CCA; pCCA, perihilar CCA; SMAD 4, SMAD family member 4.

### 3.7 Expression of STING1 and SMAD4 are independent prognostic factors of CCA

Univariate and multivariate analyses were performed to ascertain the independent prognostic factors of CCA (Tables 3, 4, 5). Factors such as N stage, STING1 expression, SMAD4 expression, and the co‐absence of STING1 and SMAD4 expression were determined as prognostic factors for intrahepatic CCA (iCCA) (Table 3). However, subsequent multivariate analysis did not validate any of these as independent prognostic markers for iCCA. In the case of perihilar CCA (pCCA), tumour size, tumour differentiation, STING1 expression, SMAD4 expression, and the co‐absence of STING1 and SMAD4 expression were identified as prognostic factors (Table 4). Further multivariate analysis has confirmed this tumour differentiation and STING1 expression as the independent prognostic biomarker of pCCA. For dCCA, univariate analysis has identified N stage, STING1 expression, SMAD4 expression and double‐negative expression of STING1 and SMAD4 as prognostic factors (Table 5), and further multivariate analysis has confirmed N stage and STING1 expression as the independent prognostic biomarker.

**TABLE 3:** The prognostic significance of clinicopathological characteristics in iCCA.

Characteristics	3‐year OS	*p* ^a^	HR	95% CI	*p* ^b^
Age (years)		0.986	–	–	–
<65	29.2		–	–	–
≥65	28.3				
Gender		0.864	–	–	–
Female	34.8		–	–	–
Male	12.2				
Tumour size		0.676	–	–	–
<2.5 cm	19.6		–	–	–
≥2.5 cm	25.5				
Differentiation		0.792	–	–	–
Well/moderate	30.4		–	–	–
Poor	0.0				
T stage		0.380	–	–	–
T1 + T2	25.4		–	–	–
T3 + T4	0.0				
N stage		**0.044**	1	–	–
N0	34.8		2.083	0.85–5.10	0.108
N1 + N2	0.0				
M stage		0.286	–	–	–
M0	27.6		–	–	–
M1	0.0				
TNM stage		0.08	–	–	–
I + II	38.5				
III + IV	12.8		–	–	–
STING1		**0.023**	–	–	–
Low	17.5				
High	28.1		–	–	0.939
SMAD 4		**0.005**	–	–	–
Low	12.8				
High	41.5		–	–	0.859
STING1+ SMAD4		**0.032**	–	–	–
Co‐expression	27.9		–	–	0.897
Other	17.9				

*Note*: The bolded *p*‐value was less than 0.05, indicating that this clinical factor was associated with the adverse prognosis of patients with CCA and had statistical significance. The bolded *p*‐value is less than 0.05, which means that this factor can be used as an independent risk factor for patient prognosis and has statistical significance.

Abbreviations: CI, confidence interval; HR, hazard ratio; OS, overall survival.

^a^ Calculated by logrank test.

^b^ Calculated by Cox‐regression Hazard model.

**TABLE 4:** The prognostic significance of clinicopathological characteristics in pCCA.

Characteristics	3‐year OS	*p* ^a^	HR	95%CI	*p* ^b^
Age (years)		0.126	–	–	–
<65	9.1				
≥65	18.9		–	–	–
Gender		0.801	–	–	–
Female	15.1				
Male	15.5				
			–	–	–
Tumour size		**0.026**	1	–	–
<2.5 cm	24.6				
≥2.5 cm	0.0		1.434	0.80–2.56	0.223
Differentiation		**<0.001**	1	–	–
Well/Moderate	22.7				
Poor	6.0		3.495	2.01–6.07	**<0.001**
T stage		0.075	–	–	–
T1 + T2a	26.0				
T2b + T3 + T4	10.9		–	–	–
N stage		0.160	–	–	–
N0	21.2				
N1 + N2	19.5		–	–	–
M stage		0.214	–	–	–
M0	20.4		–	–	–
M1	0.0				
TNM stage		0.296	**–**	–	–
I + II	21.1		–	–	–
III + IV	19.6				
STING1		**<0.001**	1	–	–
Low	6.6		0.366	0.22–0.61	**<0.001**
High	28.4				
SMAD 4		**<0.001**	1	–	–
Low	9.2		0.582	0.22–1.52	0.268
High	24.7				
STING1 + SMAD4		**<0.001**	1	–	**–**
Co‐expression	39.6		1.400	0.35–5.67	0.637
Other	10.4				

*Note*: The bolded *p*‐value was less than 0.05, indicating that this clinical factor was associated with the adverse prognosis of patients with CCA and had statistical significance. The bolded *p*‐value is less than 0.05, which means that this factor can be used as an independent risk factor for patient prognosis and has statistical significance.

Abbreviations: CI, confidence interval; HR, hazard ratio; OS, overall survival.

^a^ Calculated by logrank test.

^b^ Calculated by Cox‐regression Hazard model.

**TABLE 5:** The prognostic significance of clinicopathological characteristics in dCCA.

Characteristics	3‐year OS	*p* ^a^	HR	95%CI	*p* ^b^
Age (years)		0.556	–	–	–
<65	15.5				
≥65	32.6		–	–	–
Gender		0.412	–	–	–
Female	26.2		–	–	–
Male	28.5				
Tumour size		0.921	–	–	–
<2.5 cm	26.9		–	–	–
≥2.5 cm	28.0				
Differentiation		0.705	–	–	–
Well/moderate	29.7				
Poor	00.0		–	–	–
T stage		0.745	–	–	–
T1 + T2	19.2		–	–	–
T3 + T4	29.7				
N stage		**0.017**	1	–	–
N0	29.8		1.926	1.07	**0.029**
N1 + N2	16.8				
M stage		0.772	–	–	–
M0	29.4		–	–	–
M1	0.0				
TNM stage		0.419	–	–	–
I + II	24.5				
III + IV	35.1		–	–	–
STING1		**0.002**	1	–	–
Low	16.0				
High	43.5		0.465	0.28	**0.004**
SMAD4		**0.005**	1	–	–
Low	24.7				
High	36.9		0.509	0.21	0.128
STING1+ SMAD4		**0.010**	1	–	–
Co‐expression	36.7		1.554	0.46	0.478
Other	26.2				

*Note*: The bolded *p*‐value was less than 0.05, indicating that this clinical factor was associated with the adverse prognosis of patients with CCA and had statistical significance. The bolded *p*‐value is less than 0.05, which means that this factor can be used as an independent risk factor for patient prognosis and has statistical significance.

Abbreviations: CI = confidence interval; HR = hazard ratio; OS = overall survival.

^a^ Calculated by logrank test.

^b^ Calculated by Cox‐regression Hazard model.

## 4 DISCUSSION

The malignant degree of CCA is high, and the prognosis is poor.^15^ Currently, the treatment selections for CCA are limited, and new treatment methods are urgently needed.^5^ Immunotherapy is a therapeutic method developed in recent years that has achieved a satisfying therapeutic effect in solid tumours, including hepatocellular carcinoma,^31^, ^32^, ^33^ lung cancer,^34^ melanoma,^35^ etc. Despite efforts to employ immunotherapy as a stand‐alone treatment or in combination approaches, a significant breakthrough in the treatment of CCA using immunotherapy has yet to be achieved. The whole exon sequencing data showed that the mutation rate of tumour suppressor genes, including SMAD4 and p53, was high in CCA.^8^, ^9^, ^35^ These genetic mutations can induce genomic instability in tumour cells and are linked to an increased tumour mutation burden. However, there is currently no evidence to suggest that SMAD4 gene mutations are specifically associated with the response to tumour immunotherapy. Previous studies have shown that SMAD4 gene mutation can increase the immunogenicity of pancreatic cancer through the tumour's endogenous DNA sensing system.^36^ Therefore, we propose a hypothesis that the low responsiveness of CCA with SMAD4 mutation to immunotherapy may be caused by the absence of its endogenous DNA sensing system. To investigate the aforementioned hypothesis, we initially examined the expression levels of SMAD4 and STING1 using data from the Llovet database. The findings revealed a reduction in the expression of both SMAD4 and STING1 in tumour samples, along with a significant correlation between the expressions of SMAD4 and STING1. We further detected the expression of SMAD4 and STING1 at the protein level in our TMA. The results demonstrated a lower expression of both SMAD4 and STING1 in CCA compared to adjacent normal CCA tissues. Furthermore, there was a correlation between the expression of SMAD4 and STING1. These findings suggest that SMAD4 deletion in CCA may decrease its immunogenicity by reducing the expression of STING1.

Furthermore, we investigated the impact of SMAD4 and STING1 downregulation on the prognosis of CCA patients. Our findings reveal that decreased expression of SMAD4 is associated with a poorer prognosis, consistent with previous studies. Additionally, low expression of STING1 is also linked to an unfavourable prognosis in CCA. Notably, low expression of STING1 independently serves as a risk factor for poor prognosis in these patients. Further studies showed that the simultaneous low expression of STING1 and SMAD4 was associated with a worse prognosis. These results further suggest that STING1 and SMAD4 have synergistic effects.

In our study, we aimed to investigate the underlying mechanism behind the limited effectiveness of immunotherapy in patients with SMAD4 deficiency. However, it is important to note that this aspect of our exploration lacks further validation through cell and animal experiments. In addition, by adjusting the expression of STING1, combined immunotherapy may improve the therapeutic effect of CCA, which is worthy of further exploration.

In conclusion, our study has provided evidence that the expression of both STING1 and SMAD4 is downregulated in CCA tumour tissues when compared to the corresponding para‐tumour tissues. Additionally, we observed a positive correlation between STING1 and SMAD4 expression in CCA. Low levels of both STING1 and SMAD4 have been associated with a poor prognosis in CCA, and their simultaneous low expression is indicative of even worse patient survival. These findings suggest that the silencing or mutation of SMAD4 in CCA cells may reduce their immunogenicity and enhance their resistance to immunotherapy by suppressing the endogenous cGAS‐STING1 sensing system.

## AUTHOR CONTRIBUTIONS

**An‐da Shi:** Data curation (equal); formal analysis (equal); investigation (equal); methodology (equal). **Liming Zhao:** Conceptualization (equal); data curation (equal); formal analysis (equal). **Guoli Sheng:** Conceptualization (equal). **Ge‐ning Zhang:** Supervision (equal); writing – review and editing (equal). **Yongchang Tang:** Formal analysis (equal). **Kang‐shuai Li:** Methodology (lead); writing – original draft (lead); writing – review and editing (lead). **Zong‐Li Zhang:** Funding acquisition (lead); investigation (lead); project administration (lead).

## FUNDING INFORMATION

This work was supported by the National Natural Science Foundation of China (Grant No. 81900728, 82072676, 82172791, 82203766), Shandong Province Natural Science Foundation (Grant No. ZR2019MH008, ZR2020MH238, ZR2021QH079), Shandong Province Key R&D Program (Major Scientific Innovation Projects, 2021CXGC011105), Shandong Medical and Health Technology Development Project (Grant No. 2018WSB20002), Clinical Research Foundation of Shandong University (Grant No. 2020SDUCRCA018), Key Research and Development Program of Shandong Province (Grant No. 2019GSF108254). The funders had no role in study design, data collection, analysis, interpretation and manuscript writing.

## CONFLICT OF INTEREST STATEMENT

The authors declare no competing interests.

## Supporting information


Table S1.

## Data Availability

The data that support the findings of this study are openly available in GEO database, reference number [GSE236894].
