# Supplementary material for: SMAD4 regulates the progression of cholangiocarcinoma by modulating the expression of STING1
Source: J Cell Mol Med. 2023 Jul 24;27(17):2547–61. doi: 10.1111/jcmm.17857 (PMC10468663; doi:10.1111/jcmm.17857)
Supplement: Supplementary file 1 — Table S1. [file JCMM-27-2547-s001.docx]

**Supplemental table 1. The information of patients with iCCA of TMA.**

| **No.** | **Characteristics** | | **No.** | **Characteristics** | |
| --- | --- | --- | --- | --- | --- |
|  | **Age (years)** | **Gender** |  | **Age (years)** | **Gender** |
| 1 | 53 | male | 26 | 60 | male |
| 2 | 68 | male | 27 | 55 | female |
| 3 | 57 | female | 28 | 73 | male |
| 4 | 65 | male | 29 | 58 | female |
| 5 | 45 | male | 30 | 73 | male |
| 6 | 58 | male | 31 | 55 | male |
| 7 | 46 | female | 32 | 52 | female |
| 8 | 70 | male | 33 | 65 | female |
| 9 | 63 | male | 34 | 49 | female |
| 10 | 78 | female | 35 | 62 | female |
| 11 | 60 | male | 36 | 57 | male |
| 12 | 64 | female | 37 | 73 | male |
| 13 | 70 | male | 38 | 72 | female |
| 14 | 62 | male | 39 | 49 | male |
| 15 | 49 | male | 40 | 65 | male |
| 16 | 60 | male | 41 | 61 | female |
| 17 | 53 | female | 42 | 60 | male |
| 18 | 53 | male | 43 | 65 | male |
| 19 | 48 | male | 44 | 52 | male |
| 20 | 59 | male | 45 | 53 | male |
| 21 | 63 | female | 46 | 44 | male |
| 22 | 75 | female | 47 | 56 | male |
| 23 | 63 | male | 48 | 60 | male |
| 24 | 66 | male | 49 | 57 | male |
| 25 | 57 | male | 50 | 66 | male |

**Abbreviations:**

iCCA=intrahepatic cholangiocarcinoma

**Supplemental table 2. The information of patients with pCCA of TMA.**

| **No.** | **Characteristics** | | **No.** | **Characteristics** | |
| --- | --- | --- | --- | --- | --- |
|  | **Age (years)** | **Gender** |  | **Age (years)** | **Gender** |
| 1 | 68 | male | 35 | 67 | male |
| 2 | 62 | male | 36 | 52 | female |
| 3 | 47 | female | 37 | 75 | female |
| 4 | 50 | male | 38 | 74 | male |
| 5 | 57 | female | 39 | 78 | male |
| 6 | 70 | male | 40 | 58 | female |
| 7 | 76 | female | 41 | 68 | female |
| 8 | 75 | female | 42 | 33 | female |
| 9 | 63 | male | 43 | 77 | male |
| 10 | 55 | male | 44 | 64 | female |
| 11 | 73 | male | 45 | 56 | male |
| 12 | 55 | male | 46 | 65 | male |
| 13 | 72 | male | 47 | 65 | male |
| 14 | 64 | male | 48 | 53 | female |
| 15 | 61 | male | 49 | 58 | male |
| 16 | 49 | male | 50 | 67 | female |
| 17 | 70 | male | 51 | 66 | male |
| 18 | 48 | male | 52 | 66 | male |
| 19 | 70 | female | 53 | 65 | male |
| 20 | 63 | male | 54 | 51 | male |
| 21 | 58 | male | 55 | 64 | male |
| 22 | 76 | female | 56 | 59 | female |
| 23 | 62 | female | 57 | 75 | male |
| 24 | 62 | female | 58 | 64 | male |
| 25 | 65 | female | 59 | 53 | male |
| 26 | 41 | male | 60 | 72 | female |
| 27 | 59 | female | 61 | 63 | male |
| 28 | 49 | female | 62 | 60 | male |
| 29 | 62 | male | 63 | 69 | male |
| 30 | 66 | male | 64 | 61 | male |
| 31 | 55 | male | 65 | 81 | male |
| 32 | 34 | female | 66 | 64 | male |
| 33 | 69 | female | 67 | 64 | male |
| 34 | 62 | male | 68 | 67 | male |

| **No.** | **Characteristics** | | **No.** | **Characteristics** | |
| --- | --- | --- | --- | --- | --- |
|  | **Age (years)** | **Gender** |  | **Age (years)** | **Gender** |
| 69 | 75 | male | 92 | 62 | male |
| 70 | 72 | male | 93 | 53 | female |
| 71 | 66 | male | 94 | 54 | female |
| 72 | 62 | male | 95 | 62 | male |
| 73 | 70 | male | 96 | 64 | male |
| 74 | 46 | male | 97 | 75 | male |
| 75 | 63 | female | 98 | 55 | female |
| 76 | 66 | male | 99 | 56 | male |
| 77 | 61 | female | 100 | 56 | female |
| 78 | 66 | male | 101 | 71 | male |
| 79 | 65 | female | 102 | 38 | male |
| 80 | 60 | male | 103 | 64 | male |
| 81 | 49 | female | 104 | 63 | female |
| 82 | 68 | male | 105 | 74 | female |
| 83 | 58 | female | 106 | 49 | male |
| 84 | 68 | female | 107 | 72 | male |
| 85 | 70 | male | 108 | 76 | female |
| 86 | 56 | male | 109 | 64 | female |
| 87 | 70 | male | 110 | 63 | female |
| 88 | 57 | male | 111 | 65 | male |
| 89 | 67 | male | 112 | 58 | male |
| 90 | 65 | male | 113 | 64 | male |
| 91 | 69 | male |  |  |  |

**Abbreviations:**

pCCA=perihilar cholangiocarcinoma

**Supplemental table 3. The information of patients with dCCA of TMA.**

| **No.** | **Characteristics** | | **No.** | **Characteristics** | |
| --- | --- | --- | --- | --- | --- |
|  | **Age (years)** | **Gender** |  | **Age (years)** | **Gender** |
| 1 | 71 | female | 35 | 62 | male |
| 2 | 68 | female | 36 | 62 | male |
| 3 | 70 | male | 37 | 67 | female |
| 4 | 68 | male | 38 | 72 | female |
| 5 | 68 | male | 39 | 63 | male |
| 6 | 55 | male | 40 | 69 | female |
| 7 | 64 | male | 41 | 67 | male |
| 8 | 78 | male | 42 | 63 | female |
| 9 | 46 | female | 43 | 51 | female |
| 10 | 66 | male | 44 | 69 | male |
| 11 | 66 | male | 45 | 46 | female |
| 12 | 72 | male | 46 | 50 | male |
| 13 | 42 | male | 47 | 62 | female |
| 14 | 74 | male | 48 | 67 | female |
| 15 | 54 | male | 49 | 76 | male |
| 16 | 70 | male | 50 | 62 | male |
| 17 | 81 | male | 51 | 70 | female |
| 18 | 61 | male | 52 | 62 | male |
| 19 | 55 | female | 53 | 68 | female |
| 20 | 80 | male | 54 | 64 | female |
| 21 | 45 | male | 55 | 66 | female |
| 22 | 69 | male | 56 | 41 | female |
| 23 | 71 | female | 57 | 51 | female |
| 24 | 56 | male | 58 | 77 | male |
| 25 | 57 | male | 59 | 63 | female |
| 26 | 81 | female | 60 | 77 | female |
| 27 | 78 | male | 61 | 53 | male |
| 28 | 58 | male | 62 | 62 | female |
| 29 | 62 | male | 63 | 54 | male |
| 30 | 60 | male | 64 | 66 | male |
| 31 | 53 | male | 65 | 75 | male |
| 32 | 63 | female | 66 | 76 | male |
| 33 | 76 | male | 67 | 63 | male |
| 34 | 65 | male | 68 | 60 | male |

| **No.** | **Characteristics** | | **No.** | **Characteristics** | |
| --- | --- | --- | --- | --- | --- |
|  | **Age (years)** | **Gender** |  | **Age (years)** | **Gender** |
| 69 | 60 | male | 95 | 78 | male |
| 70 | 59 | male | 96 | 47 | male |
| 71 | 55 | male | 97 | 64 | male |
| 72 | 56 | male | 98 | 79 | male |
| 73 | 50 | male | 99 | 73 | male |
| 74 | 44 | male | 100 | 74 | male |
| 75 | 86 | male | 101 | 55 | female |
| 76 | 57 | female | 102 | 65 | female |
| 77 | 53 | male | 103 | 54 | male |
| 78 | 79 | male | 104 | 58 | female |
| 79 | 67 | male | 105 | 71 | male |
| 80 | 63 | female | 106 | 68 | male |
| 81 | 59 | male | 107 | 65 | male |
| 82 | 56 | female | 108 | 63 | male |
| 83 | 55 | male | 109 | 63 | male |
| 84 | 67 | female | 110 | 64 | male |
| 85 | 66 | male | 111 | 56 | male |
| 86 | 59 | male | 112 | 56 | female |
| 87 | 65 | male | 113 | 36 | female |
| 88 | 64 | male | 114 | 54 | male |
| 89 | 74 | male | 115 | 37 | male |
| 90 | 63 | female | 116 | 67 | male |
| 91 | 62 | male | 117 | 66 | male |
| 92 | 60 | female | 118 | 74 | male |
| 93 | 68 | male | 119 | 55 | male |
| 94 | 60 | female |  |  |  |

**Abbreviations:**

dCCA=distal cholangiocarcinoma
